# Risk assessment of glyphosate and malathion pollution and their potential impact on *Oreochromis niloticus*: role of organic selenium supplementation

**DOI:** 10.1038/s41598-022-13216-y

**Published:** 2022-06-15

**Authors:** Marwa A. Hassan, Samaa T. Hozien, Mona M. Abdel Wahab, Ahmed M. Hassan

**Affiliations:** 1 Department of Animal Hygiene, Zoonoses and Behavior, Faculty of Veterinary Medicine, Suez Canal University, Ismailia, 41522 Egypt; 2 Animal Health Research Institute, Ismailia, 41522 Egypt

**Keywords:** Physiology, Environmental sciences

## Abstract

A field survey was conducted on five fish farms to trace glyphosate and malathion pollution with some physicochemical parameters. A precise half-life time, LC_50_-96h, of these agrochemicals on *Oreochromis niloticus*, as well as chronic exposure with organic selenium (OS) supplementation, were experimentally investigated. *Oreochromis niloticus* was subjected to the following: (negative control); (2 mg L^−1^ glyphosate); (0.5 mg L^−1^ malathion); (glyphosate 1.6 mg L^−1^ and 0.3 mg L^−1^ malathion); (glyphosate 2 mg L^−1^ and OS 0.8 g kg^−1^ diet); (malathion 0.5 mg L^−1^ and OS 0.8 g kg^−1^ diet) and (glyphosate 1.6 mg L^−1^; malathion 0.3 mg L^−1^ and OS 0.8 g kg^−1^ diet). Furthermore, data from the analyzed pond revealed a medium risk quotient (RQ) for both agrochemicals. The detected agrochemicals were related to their application, and vegetation type surrounding the farms, also their biodegradation was correlated to water pH, temperature, and salinity. Glyphosate and malathion had half-lives of 2.8 and 2.3 days and LC_50_-96h of 2.331 and 0.738 mg L^−1^, respectively. The severest nervous symptoms; increased oxidative stress markers, as well as high bacterial count in the livers and kidneys of fish challenged with Aeromonas hydrophila, were observed in the combined exposure, followed by a single exposure to malathion and then glyphosate. Organic selenium mitigated these impacts.

## Introduction

Egypt was ranked seventh among the top global aquaculture producers in 2018^1^, aquaculture production increased from 18.5% in 1992, to 81% of total production in 2018^1,2^. Egypt is currently Africa's leading producer^3^ as well as the world's third-largest tilapia producer. Due to water limitations, employing agricultural drainage water for aquaculture in Egypt is challenging^4^. According to Law No. 124 of 1983, which prohibits the use of freshwater for fish culture, fish farming in Egypt is reliant on drainage water^5^. As a result, over 90% of fish farms rely mostly on agricultural drainage waters^6^. The risks of drainage water have escalated in recent years because fish farm irrigation in a nearby water body is fully dependent on drainage water^5^. This drainage water was not pre-treated, and it contained around 370 different organic and inorganic constituents, including potentially hazardous elements (PHEs)^7^.

Pesticides are widely used in agricultural watersheds, polluting a variety of aquatic environments^8^, they continue to be a source of environmental concern due to their continual release^4^. They may build in high concentrations in aquatic systems as spray drift and soil leaching, threatening aquatic animal populations^9^. In addition to their toxicity and environmental endurance, they can accumulate significantly in aquatic animal’s tissue throughout the food chain, posing a significant public health hazard^10^. Organophosphate (OP) has been chosen as today's most preferred insecticide to increase the productivity of pest-free crops USEPA^11^, consequently, they have a significant impact on the health as well as metabolic processes of tilapia fish, which is considered an essential commercial protein source in Egypt^12^. Shalaby, et al.^13^ detected significant levels of total organophosphorus pesticide residues in water (73.57 ± 62.97 μg L^−1^), sediment (103.03 ± 16.05 ppb), and fish muscle samples (*Claris gariepinus* 49.1 ± 17.8 μg L^−1^, *Tilapia zilli* 48.3 ± 18.9 μg L^−1^ and *Oreochromis niloticus* (45.6 ± 28.7 μg L^−1^) in samples collected from Nile river, Cairo, Egypt. Furthermore, significant quantities of malathion, diazinon, chlorpyrifos, cadusafos, and prothiphos were reported in various Nile River tributaries in Egypt^14^.

Regular monitoring program for pesticides is desperately needed to assess these risks associated with such pollutants^15^.The Environmental Protection Agency (EPA) model creates a peak expected environmental concentration (EEC)^16^ of a targeted pesticide in a defined natural aquatic waterbody at a specified distance from the site of application^17^. In human health and environmental risk evaluations, EECs of a wide range of compounds, including some that are not insecticides, are employed^18^. EECs are assessed against toxicity values (LC_50_) in risk assessment to define the probability of toxicity at a particular dose of exposure^16,19^. The evaluation of EECs in this manner contributes to the establishment of environmental standards, strategies, regulations, and restrictions, and also the certification of chemicals for legal use^16^. Furthermore, the Risk Quotient (RQ) method is frequently used in the assessment of the environmental risk of pesticides and other chemicals^20^, which is primarily used by the US EPA to assess the ecological risk of pesticides quantitatively by measuring the ratio of a predicted value of exposure to a predicted value of effects.

The International Agency for Research on Cancer as well as the World Health Organization (WHO) classified glyphosate (organophosphate insecticide), malathion and diazinon (organophosphate insecticides) as "possibly carcinogenic to humans" (Group 2A). Moreover, these two pesticides and glyphosate are registered and frequently used in the Egyptian market^21^. Glyphosate, a herbicidal active substance (AS), is the world's best-selling chemical herbicide and is found in a wide range of pesticide products (glyphosate-based herbicides; GBH)^22^. Glyphosate traces have been identified in surface waterways in various sites (8.7 μg L^−1^^23^, 86 μg L^−1^^24^, and 430 μg L^−1^^25^). Glyphosate can act synergistically with parasites to reduce fish survival when found in ecosystems at realistic concentrations^26^. Glyphosate and or GBHs in non-targeted species may be directly or indirectly associated with the generation of oxidative stress^27^ via induction hepatotoxicity in fish by the involvement of oxidative stress, inflammatory response, and lipid metabolism disorder, all of which were intimately interrelated during glyphosate exposure^28^. Malathion, one of the first organophosphate insecticides produced in the 1950s, was initially authorized for use in the United States by the United States Department of Agriculture (USDA) in 1956 and is presently governed by the USEPA^11^. It is one of the most effective that is commonly used in agriculture to control a wide range of insects, hence it has the potential to enter aquatic habitats and impact non-target species such as fish^29^. When it is released into the environment, it is known to cause severe metabolic disorders in non-target species like fish and freshwater mussels^11^. Malathion inhibits the acetylcholinesterase enzyme and prevent the hydrolysis of the neurotransmitter acetylcholine in the central and peripheral nervous systems^30^, in addition to disruption of normal cellular function by causing oxidative stress^31^. Malathion residue in some water samples has been detected at different localities in Egyptian governorates (Cairo 1.76 μg L^−1^, Alexandria 7.63 μg L^−1^, Damietta 5.73 μg L^−1^ and Manzla 7.63 μg L^−1^)^15^.

Selenium (Se) is an essential trace mineral that is required for fish growth^32^. Se's primary function is to conserve biological molecules, including DNA, proteins, and lipids, while also protecting against free radicals produced by regular metabolism^33^. Se is vital for selenoprotein activity, notably Se-dependent glutathione peroxidase (GPX), which protects cells from oxidative stress^34^. This complex, which is composed of numerous isoenzymes, aids in reduction of peroxides and hydroperoxides and transforms them into more stable water and alcohols^35^. Various sources of selenium (inorganic and organic) supplemented in the diet could significantly improve GSH-Px activity and decrease mortality rate, also it could be considered a principal component of a few catalysts (glutathione peroxidase and thioredoxin), selenium had effective physiological cell reinforcement as well as tissues protection against oxidative damage^36^. Selenium supplementation in the fish diet has a considerable biochemical effect, that could enhance disease resistance and survival rate, considering it a key element in fish nutrition during intensive breeding^37^. Organic Se than inorganic Se had better absorption, tissue accumulation, and antioxidant activity, as well as lower toxicities^38^ and less environmental pollution^39^. Even though seleniumomethionine accounts for most of Se in Se-enriched yeast, dietary supplementation of 0.5–4 g kg^−1^ could provide the maximum benefits ^40^. As a result, the European Food Safety Authority (EFSA) recommends a maximum Se content in animal feeds of 0.5 mg/kg^41^ and a maximum supplementation of selenomethionine (Se-Met) as an additive of 0.2 mg/kg^41^. Moreover, Mechlaoui, et al.^42^ reported that organic selenium supplementation of up to 0.2 mg kg^−1^ (1–1.1 mg/kg assessed dietary selenium), notably in the form of hydroxy selenomethionine, OH-SeMet, had a positive effect on growth, hepatic morphology maintenance, and better protection against acute or chronic stress in juvenile gilthead seabream.

Glyphosate and malathion applications are commonly abused in Egyptian agriculture sector, inevitably threatening aquaculture and human health. Moreover, data on the combined effect of these agrochemicals on *Oreochromis niloticus* is limited. Additionally, the efficacy of organic Se sources such as selenomethionine has been investigated in several animal species, but there has been a gap in studies with Nile tilapia, particularly regarding pesticide exposure. Therefore, the objective of this study was to trace these two agrochemicals in some fish farms and assess their ecological risk, as well as their degradation, and to investigate their impact in a single or combined exposure on *Oreochromis niloticus's* behavior, oxidative stress markers, and the immunological response of fish challenged with *Aeromonas hydrophilia*. Finally, the protective role of organic selenium supplementation was evaluated.

## Methods

The current study was classified into two main sections (field survey and experimental study) as follows:

### Field survey and risk assessment of glyphosate and malathion in some fish farms

A field survey was conducted in five locations at Ismailia Governorate, Egypt, that were selected in accordance with Egyptian general authority for fishery resources development (GAFRD) regulation in suppling water for aquaculture near irrigation drainage canals (El-Salam canal latitudes, 32° 40′ to 44°, longitudes 31° 40′ to 16°) and fish farms in agricultural zones, representing different systems (water sources and fish culturing species) of fish farming (Table S1) to evaluate the hygienic quality of water with special reference to some common pollutants. Eighty-four water samples were collected from 28 fishponds (3 samples/pond) (Table S2) during June and July 2020, representing the most stressful state of water on fish health and the highest agricultural pollution with chemicals due to excessive use (glyphosate and malathion). Grab samples were collected at 30–50 cm below the water surface in the morning (9–10 AM), all precautions followed the guidelines recommended by APHA^43^. Physicochemical analysis of the collected water samples were carried out according to APHA^43^ including, the determination of temperature, pH, dissolved oxygen, and salinity (immediately measured in the site). Furthermore, nitrate, total hardness, calcium, magnesium, and total chloride were determined as quickly as possible. Both glyphosate and malathion were determined within 48 h following the APHA^43^ recommendations, and the samples were maintained in the refrigerator (4° C) until analysis, using high-performance liquid chromatography (HPLC) using the high-performance liquid chromatography (HPLC) Agilent Series 1200 quaternary gradient pump, Series 1200 autosampler, Series 1200 UV and fluorescence detector, and HPLC 2D ChemStation software (Hewlett-Packard, Les Ulis, France). The analytical column (stationary phase) was a reversed-phase C18 (250*4.6 mm, 5 μm) Teknorama (Spain).

Risk assessment was conducted by calculating the expected environmental concentration (ECC), acute effect concentration (AEC) and risk quotient (RQ) according to USEPA^16^ and EPA^44^ using the following equations:

$$\mathrm{ECC}=\left(App rate\times \frac{100}{DW+\left(Dsed\times \left(\theta +\left(P\times Kd\right)\right)\right)}\right)\times \mathrm{exp}\left(-day\times KAAM\right),$$

where DW = water depth, Dsed = sediment bulk density = 1300 kg/m^3^, $\theta$ =sediment porosity = 0.509, P = organic carbon fraction, Kd = soil: water partition coefficient, KAAM = aerobic aquatic rate constant (day^−1^), Day = time (days after application)

$$AEC=\frac{Acute \left(LC50\right) Toxicity}{10}$$

$$RQ=\frac{EC}{\mathrm{Acute }\left(\mathrm{LC}50\right)}$$

### Ethics approval and consent to participate

All procedures involving animals in this study were carried out in accordance with the Universal Directive on the Protection of Animals Used for Scientific Purposes, in accordance with ethical guidelines approved by the ethics of scientific research committee, Faculty of Veterinary Medicine, Suez Canal University, Ismailia, Egypt, and was approved by the scientific research committee (Approval number: 2016092). All methods are reported in accordance with the ARRIVE guidelines for reporting animal experiments (https://arriveguidelines.org).

## Experimental study

### Chemicals

Glyphosate (48% purity) and malathion (57% purity) were purchased from Egypt Kim International Agrochemicals and prepared with distilled water to make a stock solution to evaluate their half lifetime under the physicochemical parameters of the same water which were to be used for experimental fish exposure to elucidate acute and chronic toxicity. The half-life was determined using the dissolved chemicals in triplicate (100 L aquarium) for 72 h at a concentration of 2 mg L^−1^ glyphosate and 0.5 mg L^−1^ malathion at a water temperature of 22 ± 1 °C and pH 8 ± 0.1 (same condition of water in exposing fish to the pollutants). Water samples were collected at 24-h intervals, and concentrations were determined by HPLC using the techniques described above. The samples were processed for probit analysis to calculate the half-life and predict the degradation rate of both chemicals.

### Experimental *Oreochromis niloticus*

A total of 550 apparent healthy *Oreochromis niloticus* free from any skin lesions or microbial infections with an average body weight of 14 ± 0.5 g was obtained from nursery ponds at the Central Aquaculture Research Laboratory, Suez Canal University, Ismailia, Egypt. The fish had been acclimatized for two weeks in two fiberglass tanks, filled with aerated sterile freshwater with a holding capacity of 1000 L. The DO was maintained at ≥ 5 mg L^−1^, the water temperature at 22 ± 1 °C, and 12 h light/12 h dark, photoperiod was adopted. Ammonia (NH_3_) levels in the water were measured 3 times a week (date revealed that ammonia levels did not exceed 0.05 mg L^−1^ using spectrophotometric phenate methods^43^. To eliminate any stress on fish during changing of water, replacing of water was from the same source which was stored in a cement tank of 25 m^3^ capacity, at 15% daily as well as the recommendation of El‐Sayed^45^, and frequent siphoning of fish wastes were performed. The fish were fed daily to apparent satiety on commercial pellets of 1.5 mL (Skereting 30% protein). As recommended by AFS-FHS^46^, random fish samples were collected and subjected to bacteriological examination (liver, kidney, and spleen tissues were streaked onto Brain Heart infusion agar, (Neogen, UK) and external parasites examination (gill clipping and a skin scraping were collected and examined microscopically to reveal the presence of external parasite and other abnormalities). Only fish apparent healthy with active responses, and superior performance were selected for the challenge trial with the agrochemicals.

### Determination of lethal concentration 50 (LC_50_-96 h)

The starting concentrations used to determine LC_50_-96h were chosen based on survey results (Table S2) that aligned with ECC (Table S2) of initial pollution levels found in water samples collected from various fishponds, as well as published literature^9,47^. Two hundred and seventy *Oreochromis niloticus* were randomly assigned to 9 groups (*n* = 30/group), for each 3 glass aquariums (45 × 50 × 100 cm) of 100 L capacity were assigned (10 fish per aquarium). The first group served as a negative control, as it was not exposed to glyphosate or malathion, whereas the remaining eight groups were exposed to glyphosate (0.5, 1.5, 2, and 2.5 mg L^−1^) and malathion (0.25, 0.5, 1, and 1.5 mg L^−1^), respectively. The trial was conducted in aerated freshwater aquaria supplied with dechlorinated tap water at 22 ± 1 °C. Continuous aeration was provided to maintain water DO at a range of 5.5–6.5 mg L^−1^. The feeding was stopped 24 h before the experiment's conduction and fish was not fed during entire experimental periods as per guidelines of the Organization for Economic Cooperation and Development recommended for testing the effects of chemicals on aquatic organisms Directorate^48^. Semi-static bioassay was carried out for a period of 96 h as per standard methods^43^. Chemicals were kept almost constant considering the daily water exchange of half-life time to counterbalance decreasing pesticide concentrations. The same levels of daily water exchange were applied to the non-exposed control. Mortality was observed and recorded at 24, 48, 72, and 96 h after exposure to both chemicals, and the data were subjected to probit analysis for the calculation of the relative acute toxicity of glyphosate and malathion in a variety of concentrations on *Oreochromis niloticus* (LC_50_-96h) value.

### Fish diet preparation

For treatments supplemented with organic selenium (OS), commercial basal diet (Table S3) was crushed and mixed with 0.8 g organic selenium (*Saccharomyces cerevisiae*) per Kg of diet (YEAST SEL 2000, ULTRA BIO-LOGIC Ine) containing 2.36 mg kg^−1^ selenomethionine and 0.94 mg organic selenium)). The diet was preformed to pellets, spread to dry and stored at 4 °C for the feeding experiment. All groups were fed approximately 3% fish body weight per day twice daily.

### Experimental design and parameters measurement

**Figure 1:**
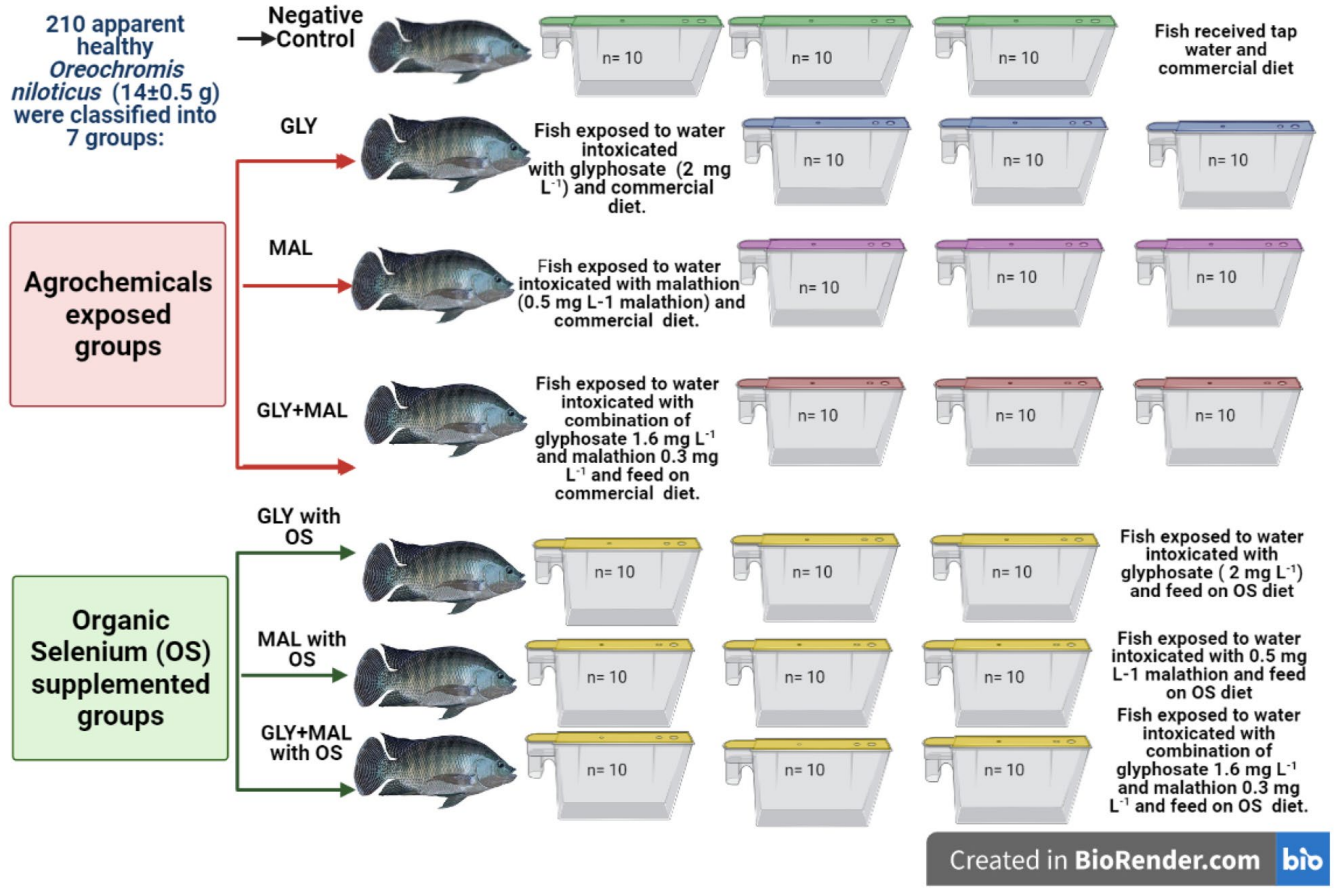
Experimental groups design illustration.

The experiment was conducted on a sublethal dose (probit analysis, Table S4) by adding LC_15_ (2 mg L^−1^ for glyphosate and 0.5 mg L^−1^ for malathion) for single pollutant exposure, and LC_1_ (1.6 mg L^−1^ for glyphosate + 0.3 mg L^−1^ for malathion) for co-exposure and shown alliance with predictions of initial pollution levels found in water from the field survey. Briefly, 210 apparent healthy fish (14 ± 0.5 g) were randomly assigned to one of seven groups (n = 30) with triplicates as described in (Fig. 1) Fish were exposed to the above-mentioned pollutant levels for a period of 60 days. Water in aquariums was completely changed almost every three days to simulate field conditions, and pesticides concentrations were adjusted with each water change. During the experimental period, The DO, pH, temperature, salinity, nitrogenous compounds, alkalinity, total hardness, and chloride were maintained within acceptable limits to avoid any stress condition correlated to water parameters to assure that any effects were due to agrochemicals as well as added organic selenium. The following parameters were measured:

Fish movement, vitality and the abnormal clinical signs in each fish aquarium were reported and cumulative survival rate was analyzed according to Kaplan and Meier^49^ and the postmortem examination of dead fish was recorded.

Samples were collected on the 30^th^, 45^th^ and 60^th^ day of the experiment (a total of 9 samples/treatment) of homogenized liver and kidney tissue samples were centrifuged at 11,000 rpm for 30 min, and the supernatant was used to determine enzyme and lipid peroxides. All treatments were carried out at 4 °C. Malondialdehyde (MDA) was assessed using the Buege and Aust^50^ method, superoxide dismutase (SOD) activity was estimated using the Paya, et al.^51^ method, and glutathione peroxidase (GPX) activity was evaluated using the modified Mills' procedure published by Hafeman, et al.^52^.

On the 60th day of the experiment, fish subjected to various treatments were experimentally challenged with *Aeromonas hydrophilia* as follows: fully identified pure *Aeromonas hydrophila* isolate with GenBank accession number OK602647.1 has been kindly provided by Professor Dr Alaa Eldin Eissa, Chair of Aquatic Animal Medicine and Management Department, Faculty of Veterinary Medicine, Cairo University. The fresh colonies were obtained, washed, and suspended in sterile normal saline (0.85% Na Cl) then matched against McFarland standard, 0.1 ml (10^5^ CFU) was injected intraperitoneal as low as a sublethal dose of bacterial suspensions 24 h bacterial culture^53^, the negative control was intraperitoneally injected with 0.1 ml normal saline and fish were observed for pathologic signs for two weeks. All fish were clinically examined for detecting abnormalities, and freshly dead and moribund fish were subjected to postmortem examination after euthanization The internal organs were exposed and examined macroscopically for any gross abnormalities. The internal organs were exposed and examined macroscopically for any gross abnormalities. Microbial colonization was evaluated by isolating and quantifying bacteria from the kidney and liver using the drop plate technique described by Herigstad et al.^54^.

### Statistical analysis

The obtained results were analyzed using the SPSS version 22 software computer program, New York, USA (Inc., 1989–2013). Principal components analysis (PCA) was used to summarize the major relationship between water quality parameters, then KMO and Bartlett’s sphericity tests were operated to verify the applicability of PCA to raw data. The correlation coefficient matrix and eigenvalues also were computed as the eigenvalue > 1.0 was accepted. A one-way analysis of variance (ANOVA) test with the least significant difference (LSD) technique was used to examine the mean differences. Probit analysis was used to determine both half-life and LC_50_ 96 h. Vitality and behavioral changes scoring were analyzed using the Friedman Test and A Kruskal–Wallis H test. The Kaplan–Meier test was performed to examine the differences between survival curves.

## Results

### Risk assessment and field survey and of glyphosate and malathion in some fish farms and half-life

Regarding risk assessment of both agrochemicals in the examined farms, results revealed that acute ECC was 0.45 mg L^−1^ and 0.27 mg L^−1^ while acute RQ indicates medium risk (0.2 and 0.38) for glyphosate and malathion, respectively (Table S2).

Both glyphosate and malathion showed an overall mean of 2.083 and 2.556 µg L^−1^, respectively excluding Farm 5 (not detected), Farm 1 had the highest mean value (5.431 ± 0.212 and 4.25 ± 0.16 µg L^−1^, respectively), and Farm 4 had the lowest mean value (0.813 ± 0.021 and 0.90 ± 0.052 µg L^−1^, respectively). The principal component analysis of both agrochemicals yielded three and four principal components (PC), with cumulative variances of 86.62 and 91.145 for glyphosate and malathion, respectively. PC1 revealed a highly significant correlation between pesticide levels and pond area as well as vegetable culture; on the other hand, the frequency of application per year revealed a weak to moderate loading. Meanwhile, an inverse relationship between agrochemical detected levels and water Total Hardness > TDS > Nitrite > pH > Nitrate > Temperature was reported (Table 1).

**Table 1:** Component matrix for different physicochemical parameters of fish farm water with glyphosate and malathion.

Chemicals	Glyphosate			Malathion			
Components	CP1	CP2	CP3	CP1	CP2	CP3	CP4
Pond depth (m)	− 0.33	0.265	0.628	− 0.301	0.167	0.71	0.565
Pond area (acre)	− 0.85	− 0.21	0.447	− 0.757	− 0.335	0.485	− 0.013
Vegetables	− 0.89	0.339	− 0.175	− 0.926	0.283	− 0.012	− 0.015
Tree	0.89	− 0.339	0.175	0.926	− 0.283	0.012	0.015
Application (ml/l)	− 0.326	0.404	0.811	0.908	− 0.271	0.167	− 0.236
Frequency/year	− 0.271	0.944	− 0.062	− 0.695	0.059	− 0.354	0.591
Glyphosate/Malathion Conc	− 0.757	− 0.009	0.501	− 0.721	0.373	0.399	− 0.396
pH	0.582	− 0.675	0.282	0.634	− 0.648	0.048	0.303
Temperature (°C)	0.401	0.853	0.033	0.333	0.874	0.069	0.092
TDS (mg L^−1^)	0.869	0.016	0.436	0.915	0.024	0.218	0.162
Total hardness (mg L^−1^)	0.913	0.07	0.324	0.951	0.094	0.153	0.056
Nitrite (NO_2_) mg L^−1^	0.823	0.122	0.141	0.82	0.195	0.1	0.041
Nitrate (NO_3_) mg L^−1^	0.606	0.701	0.071	0.574	0.747	0.138	− 0.088
Dissolved oxygen (mg L^−1^)	0.492	0.69	− 0.221	0.397	0.745	− 0.274	0.166
% Explained variance	46.724	25.493	14.404	54.416	20.425	8.818	7.486
Cumulative % of variance	46.724	72.217	86.62	54.416	74.841	83.659	91.145

PC = Principal component. Strong loading values ≥ 0.75, moderate loading values (0.5–0.75) and weak loading values (0.3–0.5).

**Figure 2:**
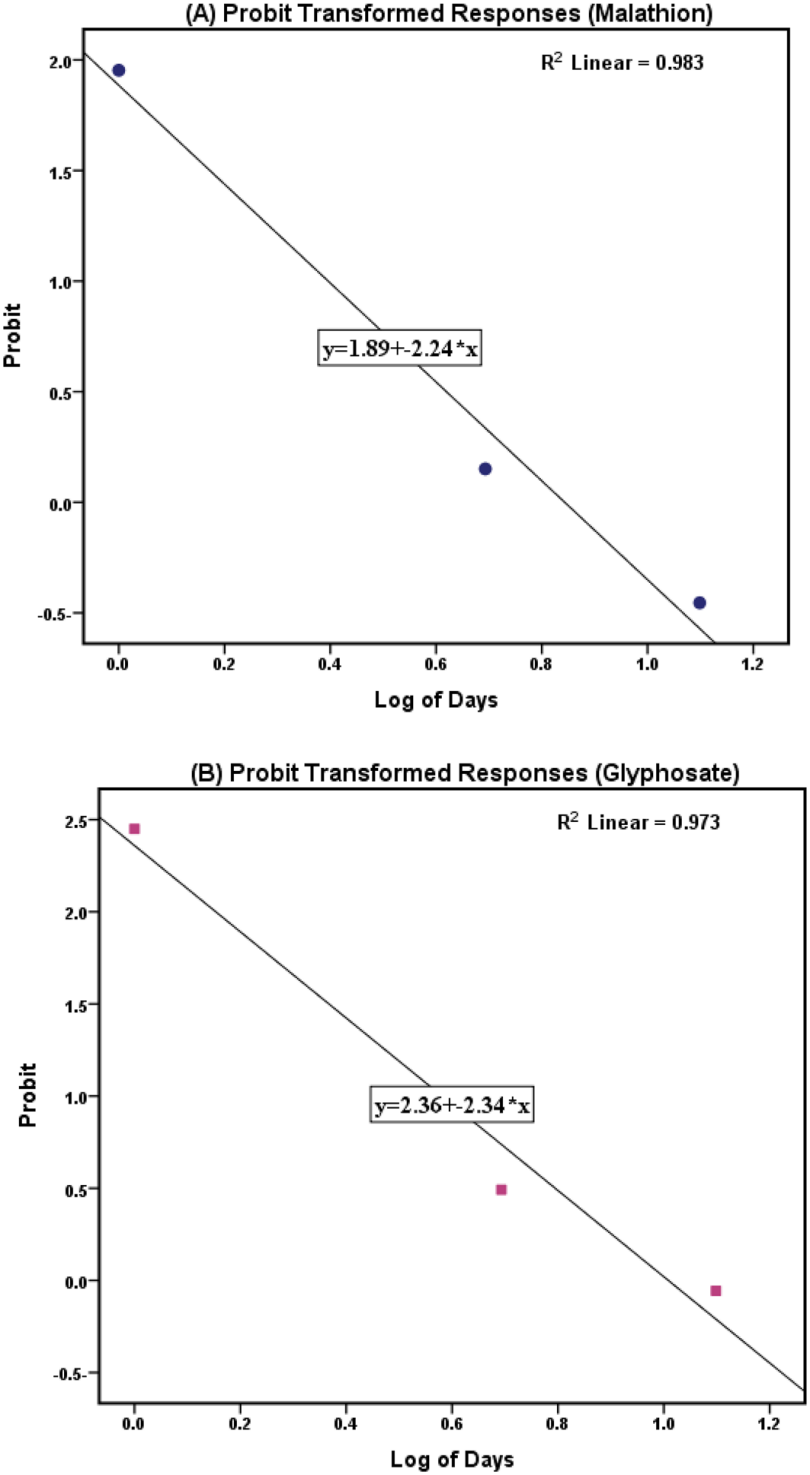
Probability for half-life of Glyphosate and Malathion mg L^−1^ in natural log days using Probit analysis.

According to probit analysis (Fig. 2), after 2.78 and 8.99 days (for glyphosate) and 2.304 and 7.003 days (for malathion), respectively, the agrochemical concentrations were expected to degrade to 50% and 0.01% of the original concentrations.

### Lethal concentration 50 (LC_50_-96h)

Table S4 shows the lethal concentrations (LC_50_) of both agrochemicals on *Oreochromis niloticus* after96 hours; the calculated LC_50_ for 96 h was (2.331 and 0.738 mg L^−1^ for glyphosate and malathion, respectively). Chronic toxicity with sublethal single exposure was induced with LC_15_ (2 mg L^−1^ glyphosate and 0.5 mg L^−1^ malathion), while LC_1_ was selected to induce chronic toxicity with combined exposure (1.6 mg L^−1^ glyphosate and 0.3 mg L^−1^ malathion).

### Fish behavior, vitality, and survival rate

**Figure 3:**
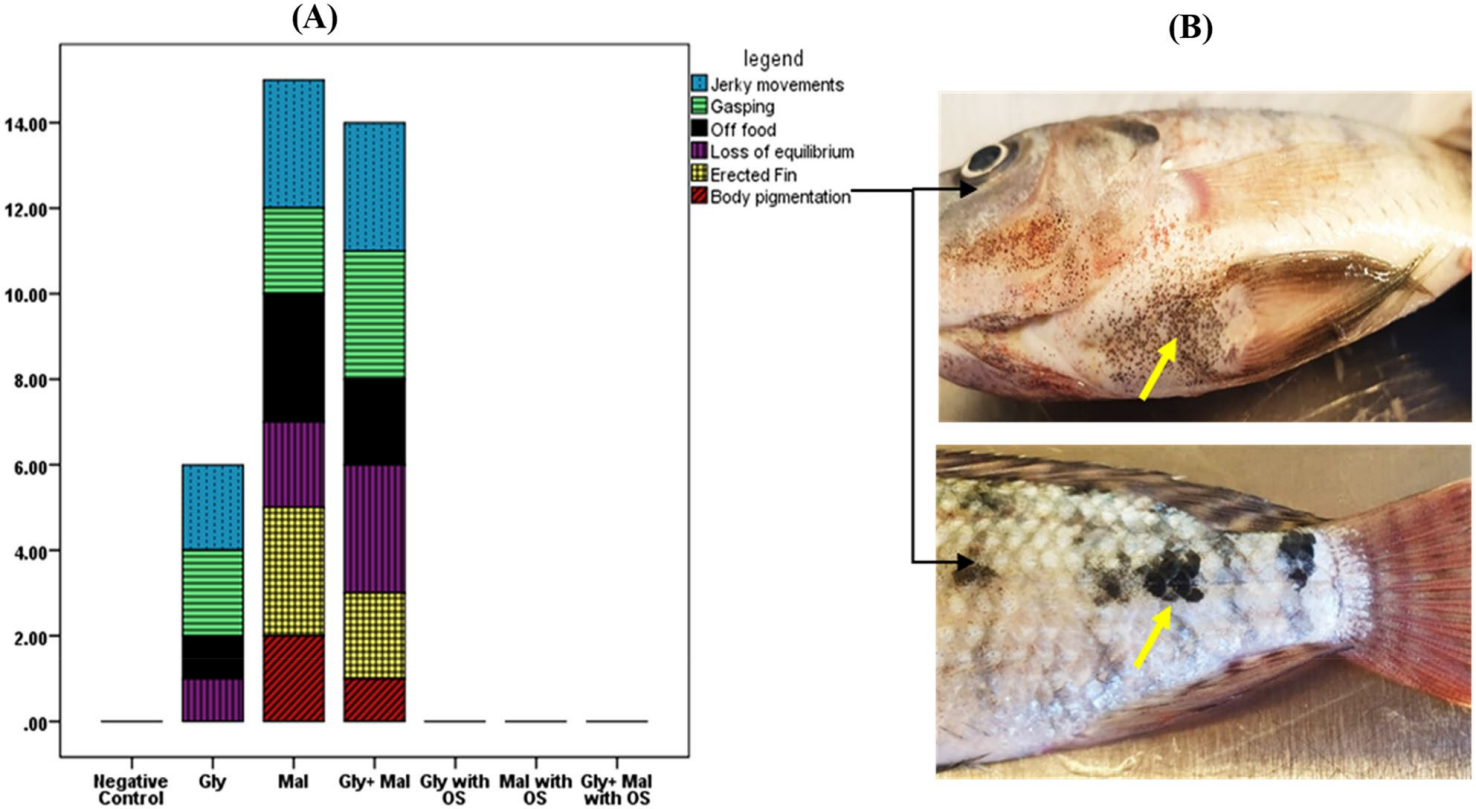
Vitality and behavioral changes scoring of *Oreochromis niloticus* exposed to glyphosate and/ or malathion with role of organic selenium. Friedman Test showed a statistically significant difference in the detected symptoms between the different treatments, χ^2^(2) = 22.75, P < 0.001, with a mean rank of 4.21, 3.79, 3.57,3.50, 3.29 and 2.64 for jerky movement, gasping, off food, loss of equilibrium, erected fin and body pigmentation. A Kruskal–Wallis H test showed that there was a statistically significant difference in the detected symptoms between the different treatments, χ^2^(2) = 20, P < 0.02, with the same mean rank score of negative control and OS treated groups in all detected signs. (**A**) Vitality and behavioral changes. (**B**) fish suffered from body pigmentation.

The stress of agrochemical exposure on fish was manifested by nervous symptoms in addition to skin changes (Fig. 3). Firstly, the detected symptoms scored using the Friedman Test showed a significant difference (P < 0.0001) between the different treatments in a descending manner, such as jerky movement, gasping, off food, loss of equilibrium, erected fin, and body pigmentation. Kruskal–Wallis H test revealed a significant difference between the different treatments, with the same mean rank score for non-exposed to chemicals negative control and organic selenium (OS) treated groups in all signs. Fish exposed to glyphosate 2 mg L^−1^ showed mild behavioral changes, ranked as follows: gasping > off food > loss of equilibrium > jerky movement, in addition to non-response when a side of the aquarium was clicked. Fish exposed to malathion 0.5 mg L^−1^ showed exaggerated symptoms with the following descending rank: off food, erected fin, skin pigmentation, and dark spots with profuse secretion of mucus after 45 days of exposure followed by jerky movement with hypersensitivity then loss of equilibrium with the sinking of the fish to the bottom and, while gasping showed lowest score. Nervous symptoms were maximum in fish exposed to glyphosate (1.6 mg L^−1^) and malathion (0.3 mg L^−1^) combination, the main manifested symptoms were gasping, and loss of equilibrium followed by jerky movement, in addition to the other symptoms. Neither behavioral nor nervous changes were observed in groups treated with organic selenium, as well as in the control group.

**Figure 4:**
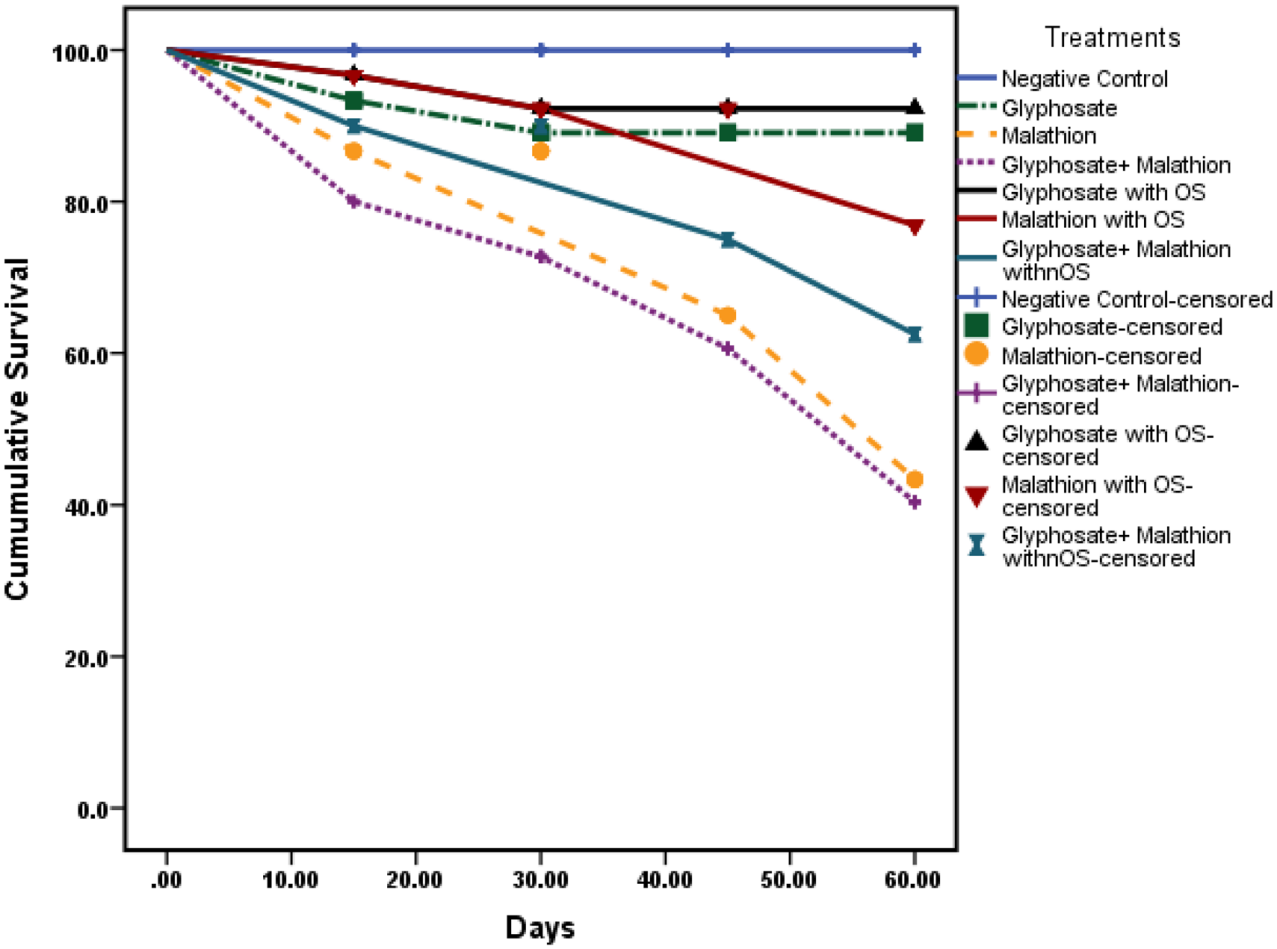
Cumulative survival rate for fish in all experimental groups exposed to glyphosate and or malathion intoxication in absent and presence of organic selenium.

It is worth reporting that the cumulative survival rate (Fig. 4) indicated that the maximum hazardous impact was reported in combined agrochemicals exposure with the lowest survival rate (60%) followed by treatment with malathion (70%), and glyphosate (80%), on the other hand, organic selenium supplementation increased the survival rate as compared to corresponding chemicals exposed groups.

### Oxidative stress markers

**Figure 5:**
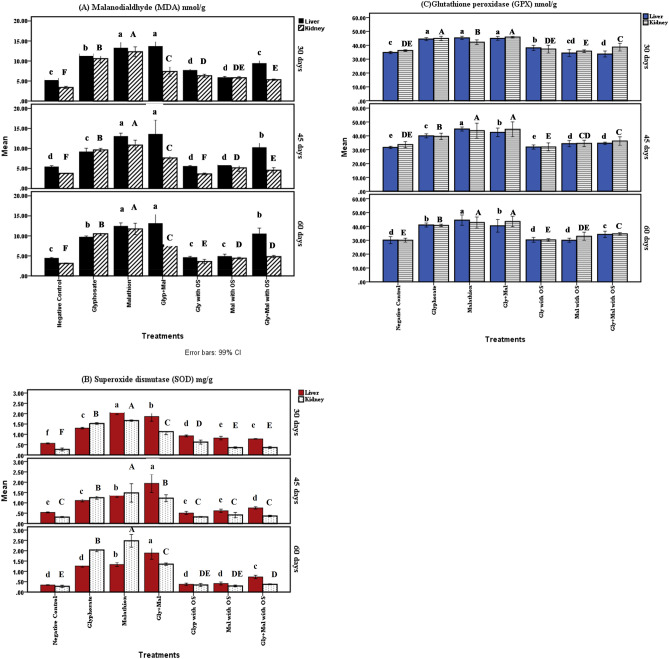
Glyphosate and /or malathion intoxication on oxidative stress biomarkers in *Oreochromis niloticus* with organic selenium supplementation. Means with different superscripts are statistically different (P < 0.05). (**A**) Malondialdehyde (MDA) in liver and kidney tissues. (**B**) Superoxide dismutase (SOD) in liver and kidney tissues. (**C**) Glutathione peroxidase (GPX) in liver and kidney tissues.

Results illustrated in Fig. 5 revealed that the values for oxidative enzyme activity (Superoxide dismutase (SOD) and glutathione peroxidase (GPX) and MDA in liver and kidney tissues elevated significantly (P < 0.05) in malathion and or glyphosate in comparison with control. Single pollutant exposure showed higher levels in fish exposed to malathion as compared to those exposed to glyphosate during the different experimental periods. OS treated groups showed a notable decrease in their levels with a non-significant (P < 0.05) difference compared to control in both tissues.

### Challenge with *Aeromonas hydrophilia*

**Figure 6:**
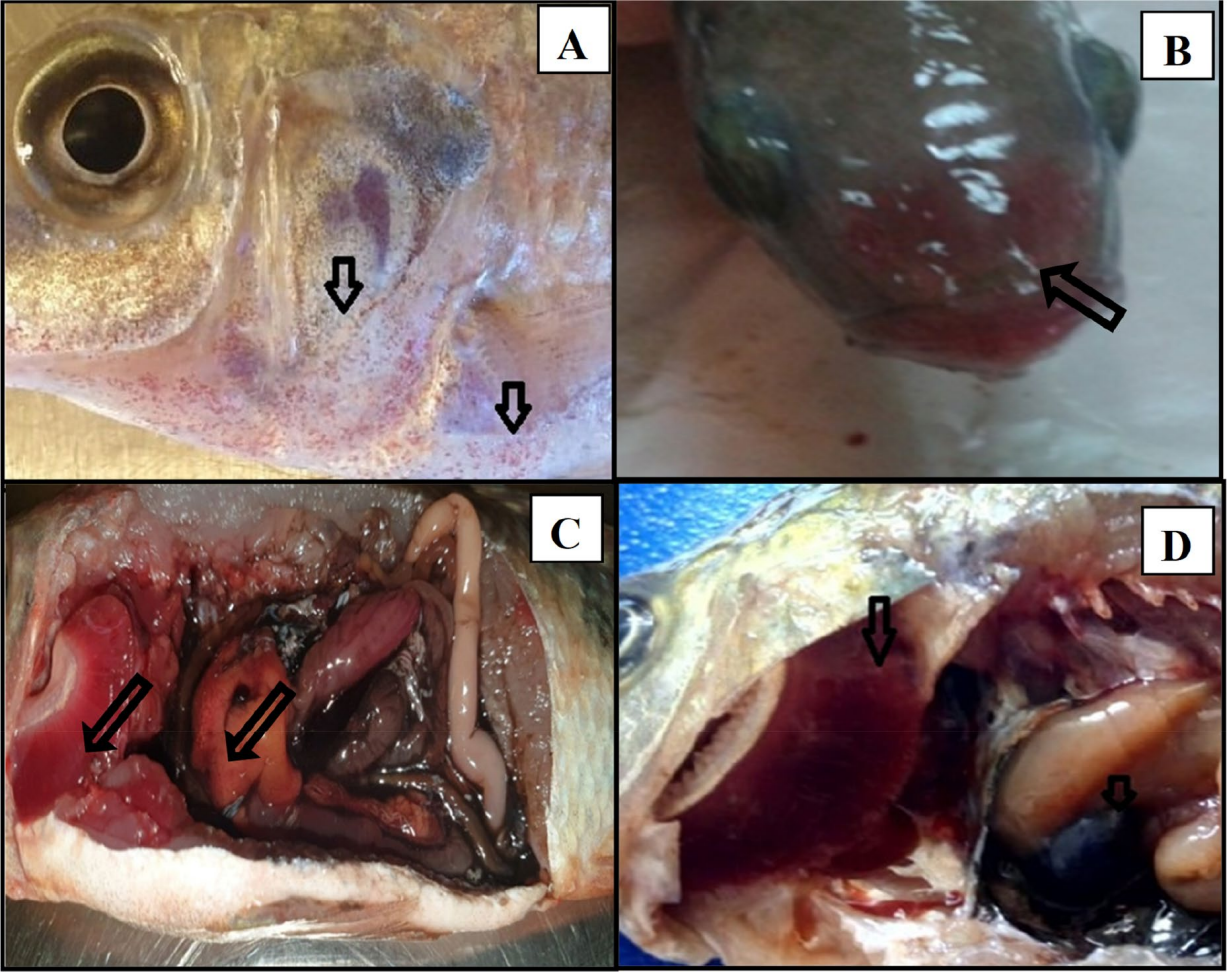
Symptoms in challenged *Oreochromis niloticus* with *Aeromonas hydrophila.* (**A**) petechial hemorrhage at pectoral fin and operculum in malathion in fish exposed to combined chemicals. (**B**) erythema and Redding of mouth in glyphosate exposed fish. (**C**,**D**) enlarged liver with congestion in gills in malathion and glyphosate exposed fish, respectively.

The observed symptoms and the postmortem examination are illustrated in Fig. 6. Mortality and *Aeromonas hydrophila* count in the livers and kidneys (log_10_ CFU mL^−1^) were higher with the highest mortality percentage and lowest relative percent survival (RPS) in the combined agrochemical exposed group. In accordance with previous results, OS supplementation not only reduced morbidity and mortality but also kept the bacterial count in the livers and kidneys low as close to the control group (Table 2).

**Table 2:** Effect of glyphosate and or malathion on fish challenged with *Aeromonas hydrophilia* and ameliorative effect of organic selenium.

Challenged groups	Relative percent survival (RPS)	Mortality	Bacterial count (log_10_ CFU/ml)	
			Liver	Kidney
NC	100	0	3.647^**d**^ ± 0.025	3.697^**d**^ ± 0.017
GLY	90.48	9.52	4.513^**b**^ ± 0.013	4.491^**b**^ ± 0.114
MAL	90.48	9.52	4.789^**a**^ ± 0.066	4.993^**a**^ ± 0.032
GLY + MAL	88.89	11.11	4.741^**a**^ ± 0.053	5.072^**a**^ ± 0.042
GLY + OS	100	0	3.816^**c**^ ± 0.021	3.742^**cd**^ ± 0.018
MAL + OS	95.24	4.76	3.734^**cd**^ ± 0.021	3.812^**cd**^ ± 0.018
GLY + MAL + OS	95.24	4.76	3.840^**c**^ ± 0.016	3.876^**c**^ ± 0.037

Means with different superscripts at the column are statistically different (P < 0.05).

**NC** (negative control); **GLY** (2 mg L^−1^ glyphosate); MAL (0.5 mg L^−1^ malathion); **GLY + MAL:** (glyphosate 1.6 mg L^−1^ and malathion 0.3 mg L^−1^). **GLY + OS**: (glyphosate 2 mg L^−1^ and Organic Selenium 0.8 g kg^−1^ diet), **MAL + OS**: (malathion 0.5 mg L^−1^ and Organic Selenium 0.8 g kg^−1^ diet) and **GLY + MAL + OS:** (glyphosate 1.6 mg L^−1^ ; malathion 0.3 mg L^−1^ and OS 0.8 g kg^−1^ diet).

## Discussion

The primary productivity and profitability of fishpond are determined by the physicochemical properties of the water used fish culture. As a result, water quality assessment is essential for developing monitoring and remediation plans to reduce the risk posed by hazardous compounds in aquatic environments^55^. The agricultural wastewater of the intensive vegetable and fruit agricultural sectors serves as the primary water source for many fish aquaculture ponds in the Nile delta's northern districts^56^. Based on our ECC and RQ analysis, glyphosate and malathion exhibited medium risk. According to Sánchez-Bayo, et al.^57^ RQ ≥ 1 represents a high risk, while 0.1 ≤ RQ ≤ 1 denotes medium risk, and 0.01 ≤ RQ ≤ 0.1 shows low risk.

In the conducted survey, both glyphosate and malathion were not detected in water samples of the farm that was supplied with underground water, which could be attributed to the fact that pesticides bind strongly to most soils and have a limited ability to migrate through the soil and pollute underground water^58^. Scientists are concerned about the presence of glyphosate in aquatic habitats because of the potential for harm to living species, such as animals and plants^59^. Obtained data revealed that the presence of glyphosate residues in water is affected by environmental factors such as pH, water depth, and water: sediment ratios^60^. The negative correlation between glyphosate concentration and hardness could be explained as hard water (high Ca^2+^ and Mg^2+^ concentrations) may influence certain herbicides by replacing the Na^+^ and K^+^ in their structures with Ca^2+^ and Mg^2^ + , changing their properties and lowering their efficacy. Similarly, an inverse relationship with pH could be due to high pH values can lead to the active principle's early breakdown^61^. Certainly, glyphosate can enter surface waterways by runoff and soil leaching, or, more rarely, through direct application into water (e.g., to manage aquatic weeds)^62^. It was obvious that the more vegetables grown, the more herbicides used to eliminate herbs inside crops, and as pond area increases, so does the surface area for glyphosate pollution arise. Despite glyphosate's strong affinity for soil particles and thus poor mobility^63^, it has been found in a variety of water bodies^62^. Generally, environmental concentrations are often in the range of a few µg L^−1^, which is substantially lower than the worst-case scenario (up to 5.4 mg µg L^−1^)^64^. However, significant concentrations have been observed in agricultural water bodies, reaching hundreds of µg L^−1^^65^. Results from previously conducted acute toxicity studies show that glyphosate and its commercial formulations are toxic at high doses, but long-term research shows that glyphosate can significantly influence the biological responses of aquatic organisms, consequently additional efforts should be directed toward assessing the chronic or sub-chronic impacts of such compounds on aquatic species^59^. The time necessary for 50% glyphosate degradation in the present study is partially in agreement with Edge et al.^60^ who found that glyphosate residues (50% degradation) in standing water depends on environmental parameters such as temperature, water depth, the presence of macrophytes, and water: sediment ratios and can range from a few days to about 4 weeks. Glyphosate evaporates quickly in moving water systems, often reaching undetectable levels in 1–4 days^66^.

Regarding malathion, Ahmed et al.^67^ reported that malathion residue levels were undetectable in shallow groundwater agricultural regions at Assuit and Sohag, Egypt. Our results revealed that malathion concentrations were significantly higher than the Environmental Protection Agency's^44^ maximum residue limits (MRLs), which set the permissible limit for malathion in aquaculture at 0.1 µg L^−1^. In addition, the obtained results are in consistence with those obtained by Derbalah and Shaheen^68^ who estimated malathion in water samples at different fish farms sites in Kafr-El-Sheikh Governorate, Egypt and found their concentration ranged from 0.37 to 4.12 μg L^−1^. Our results of malathion half-life are in consistent with findings indicating that malathion degrades faster in alkaline water, with a hydrolysis half-life of 0.2 weeks at pH 8, compared to 21 weeks at pH 6^69^. Furthermore, Keller and Ruessler^70^ demonstrated that malathion toxicity reduces with rising temperature because of increased degradation.

Although the low levels of the detected agrochemicals in the survey were less than the used concentrations to determine acute toxicity, the LC_50_-96h was based on expected environmental concentration (ECC) initial levels of pollutants. Concerning glyphosate, LC_50_-96h was higher than that obtained by Ayoola^47^ who confirmed that the LC_50_ of glyphosate was 1.05 mg/l for 96 h of exposure in the *Oreochromis niloticus* juvenile 15 ± 1 g. LC_50_ value of malathion in this study was relatively high compared to those reported by USEPA^11^ which confirmed that malathion LC_50_ in 96 h was 0.2 ppm in *Tilapia mosambica*. On the contrary, the obtained values were lower than those reported by Kandiel, et al.^71^ and Hamed^29^ who reported (LC_50_/96 h of 5 ppm and 2.71 ppm for *Oreochromis niloticus*, respectively). The difference in LC_50_ of malathion and glyphosate with other researchers could be explained in the light of prevailing water environment and fish in which the evaluation was carried out (water temperature, fish size, and type, water physical–chemical properties, any other condition that might be stressful or positive to the fish). Furthermore, differences in toxicity to different fish species may be due to differences in pesticide absorption, accumulation, biotransformation, and excretion^72^, and the magnitude of pesticide toxic effects also depends on length and weight, the corporal surface to body weight ratio, and breathing rate^73^.

The behavioral abnormalities observed in the exposed fish could be explained by the fact that organophosphorus pesticides inhibit acetylcholinesterase (AChE) and -aminolevulinate dehydratase (-ALA-D), resulting in acetylcholine accumulation, which affects neuromuscular functions (locomotion) as well as the behavior of the exposed fish^67,74,75^.

The survival rate (SR) of fish decreased with malathion treatment more than with glyphosate, since glyphosate acts mainly by inhibiting the synthesis of aromatic amino acids (phenylalanine, tyrosine, and tryptophan) through its effect on 5-enolpyruvylshikimate-3-phosphatesynthase^76^. Moreover, the effect was strengthened in combined exposure, which agreed with Laetz, et al.^77^ who reported that many insecticide combinations caused additive toxicity at low, environmentally relevant levels, also pesticide combinations demonstrated a definite pattern of synergism even at these low levels.

Following organophosphorus chemical exposure, the fish's defense mechanism attempted to fight, eliminate, or neutralize the toxic effects of reactive oxygen species (ROS) and protect the system from oxidative stress^78^. SOD activity increased significantly in fish treated with malathion and glyphosate groups, catalyzing the conversion of superoxide anion radicals (produced by malathion) to H_2_O_2_ and molecular oxygen to protect cells from oxidative damage. The method by which SOD catalyzes the conversion of superoxide anion radicals to H_2_O_2_ and molecular oxygen to protect cells from oxidative damage caused by superoxide^79^. An increase in MDA, SOD, and GPX biomarkers could be attributed to that pesticide levels used in our experiment were able to induce oxidative stress, which causes oxidative damage and a decrease in antioxidant defense^31^. *Oreochromis niloticus* exposed to malathion revealed changes in the elevation of SOD and GPX. Oxidative stress, meanwhile, resulted in a significant increase in MDA^80^.

Higher mortality was observed in fish that were challenged with *Aeromonas hydrophila* and exposed to glyphosate and or malathion. These findings confirm the stress condition induced by pesticides exposure. Other researcheindicated that glyphosate inhibited the cell-mediated immune response and reached maximum depression in tilapia after 4 weeks of exposure; it also depressed humoral immunity in a concentration-dependent pattern, and serum antibody titers in treated fish decreased in a time-dependent manner^81^. Furthermore, glyphosate exerts immune-toxic effects on common carp, causing immunological suppression or excessive activation, resulting in immune dysfunction or decreased immunity^82^. Several hematologic and immunologic blood parameters are altered in fish exposed to glyphosate, potentially leading to increased susceptibility to bacterial infections^83^.

Organic selenium supplementation to treated groups improved all measured parameters, whereas there were no behavioral or neurological abnormalities observed in groups treated with OS (0.8 g kg^−1^), which was almost comparable with the control group. It was clear that Se in the diet prevented the toxic effects of malathion by ameliorating oxidative damage and enhancing the physiological alterations which may affect the health of fish, additionally, Se had a powerful antioxidant activity and participates in the antioxidant defense system, which was involved in thyroid hormone metabolism, moderation of the immune system and prevention of cancer, acted directly as a support for the organismal health ^29,36^. Furthermore, OS stimulates AChE and mitigates the effects of pesticide exposure in *Oreochromis niloticus* by maintaining feed assimilation, metabolism, and growth^77^. Likewise, it was absorbed into the proteins of skeletal muscles, kidney, liver, and gastrointestinal mucosa as seleno-methionine and seleno-cysteine, both of which are regarded as crucial and essential micronutrients for fish^84,85^.

The current study's findings validated the role of OS in reducing oxidative stress produced by malathion and or glyphosate. As organic selenium contains antioxidant properties that boost cellular defenses against oxidative stress^86^. Generally, Se is considered a critical cell reinforcement component as it plays as a cofactor which accounted for its incorporation in selenocysteine in enzyme glutathione peroxidase (GPX), GPX scavenges H_2_O_2_ and lipid hydroperoxides, using glutathione-reducing counterparts and protecting membrane lipids and macromolecules from oxidative damage and enhancing the body’s cell resistance and that acts against reactive oxygen species (ROS)^33,87^. Se requirements in stressed fish can reach 4.0 g kg^−1^ (dry mass)^33^. Results of Se level used in our experiment are supported by other findings, whereas Dawood et al.^88^ reported that Se can protect the host cell against oxidation caused by environmental and infectious illness stressors, with the optimum quantity ranging between 0.15 and 0.7 mg kg^−1^ of diet. Kumar and Singh^89^ demonstrated that supplementation of dietary Se (1 mg kg^−1^ diet) in fish challenged with *Aeromonas sobria* resulted in lower cumulative mortality (%) and higher relative (%) survival. Se also improved the immunity of fish reared under multiple stressors and increased fish survival after infection with bacteria.

In conclusion, our study revealed that glyphosate and malathion were detected in 4 out of 5 examined fish farms, in addition, both agrochemicals had medium risk RQ. Therefore, a consistent monitoring program for fish farms is crucial for assessing the health risks associated with such pollutants. Exposure to organophosphorus agrochemicals (glyphosate 2 mg L^−1^ and malathion 0.5 mg L^−1^), and their combination (glyphosate 1.6 mg L^−1^ and malathion 0.3 mg L^−1^) negatively impacted *Oreochromis niloticus* behavioral, physiological functions, and immune response to bacterial challenge, with increasing mortality. They are considered silent killers in fish farms, resulting in severe economic losses in the aquaculture sector as they increase stress exposure, making fish farms more vulnerable to outbreaks and mortalities. The OS (0.8 g kg^−1^ of the diet containing 2.36 mg kg^−1^ selenomethionine and 0.94 mg of organic selenium) ameliorated the agrochemical detrimental effects, increasing the fish health status even with the presence of organophosphorus pesticide pollution. It is worth noting that preventing accidental or chronic pesticide exposure in fish is unachievable, thus we advise dietary inclusion of organic selenium as a sustainable bioremediation strategy, mitigating many of the impacts of glyphosate and or malathion exposure in fish.

## Supplementary Information

Supplementary Information.

## Data Availability

All data generated or analyzed during this study are included in this published article [and its supplementary information files].
