# Supplementary material for: Risk assessment of glyphosate and malathion pollution and their potential impact on Oreochromis niloticus: role of organic selenium supplementation
Source: Sci Rep. 2022 Jun 15;12:9992. doi: 10.1038/s41598-022-13216-y (PMC9200714; doi:10.1038/s41598-022-13216-y)
Supplement: Supplementary file 1 — Supplementary Information. [file 41598_2022_13216_MOESM1_ESM.docx]

**Risk assessment of glyphosate and malathion pollution and their potential impact on *Oreochromis niloticus*: Role of organic selenium supplementation**

Marwa A. Hassan^1*^, Samaa T. Hozien^2^, Mona M. Abdel Wahab^2^ and Ahmed M. Hassan^1^

^1^ Suez Canal University, Faculty of Veterinary Medicine, department of Animal Hygiene, Zoonoses and Behavior, 41522 Ismailia, Egypt

^2^Animal Health Research Institute, 41522 Ismailia, Egypt

**Corresponding author***

Marwa A Hassan

E-mail address: marwamenaem@vet.suez.edu.eg

Orchid No. https://orcid.org/0000-0001-8835-1048

**Table (S1): Topographical examination of the studied farms.**

| **5** | **4** | **3** | **2** | **1** | **Fish farm** |
| --- | --- | --- | --- | --- | --- |
| Tilapia  Mugil Cephalus | Tilapia  Mugil Cephalus | Tilapia  Mugil Cephalus Catfish | Tilapia | | **Cultured species** |
| Near agricultures | | Surrounded by agricultures | | | **Area geography** |
| Underground water | Agriculture drainage | | | | **Water source** |
| Lemon, Mango,  Olive  (trees) | Mango,  orange,  lemon  (Trees) | Mallow, Eggplant  Cucumber,  Clover  Mango, (Trees)  Palm, (Trees) | Lettuce, Tomato,  Cucumber,  Pepper,  Corn,  Pea | Eggplant,  Tomato, Cucumber,  Clover,  Cabbage | **Cultured vegetation** |
| Every 21 day (1.5 liter/ 100-liter water) | Every 21 days (1.5 liter/ 100-liter water) | Every 7 days (1 liter/100-liter water) | Every 7 days (700 ml /100-liter water) | Every 15 day (1 liter/100-liter water) | **Pesticides used:**  1-Malathion:  (throughout the year) |
| Not applied | Liter/ 50-liter water ((June) | Liter/ 100 -200-liter water  (June and July) | Liter /100-liter water  (June) | Liter/50-liter water  (June) | 2-Glyphosate  (used once or twice per year) |
| 15 sample/ 5 ponds | 18 sample/ 6 ponds | 15 sample/ 5 ponds | 15 sample/ 5 ponds | 21 sample/7 ponds | **Water samples** (sample were collected at different 3 area in each pond) |

**Table (S 2): Physicochemical parameters of water in examined fish ponds at different fish farms with risk assessment.**

| **Parameter** | **Farm 1** | **Farm 2** | **Farm 3** | **Farm 4** | **Farm 5** |
| --- | --- | --- | --- | --- | --- |
| ***Pond depth (m)** | 2.25**^a^**±0.11 | 2.167 **^a^** ±0.1 | 1.83 **^ab^** ±0.1 | 2.16**^a^** ±0.1 | 1.45**^b^** ±0.26 |
| **Pond Area (acre)** | 3 **^a^** ±0.0001 | 2**^b^** ±0.0001 | 1**^d^** ±0.0001 | 1.2**^c^** ±0.112 | 1**^d^** ±0.0001 |
| ***No. fry/pond (No.X10^4^)** | 1.4 **^ab^**±0.04 | 1.4 **^ab^**±0.04 | 1.5 **^a^** ±0.0001 | 1.25**^b^** ±0.11 | 1.5 **^a^** ±0.0001 |
| **Dissolved Oxygen (mg/l)** | 5.32**^c^** ±0.1 | 5.85**^b^** ±0.15 | 6.46 **^a^** ±0.10 | 6.31 **^a^** ±0.08 | 5.7**^b^** ±0.041 |
| **pH** | 7.64**^b^**±0.048 | 7.81**^b^**±0.117 | 6.96**^c^**±0.06 | 8.65 **^a^** ±0.13 | 8.81 **^a^** ±0.13 |
| **Temperature (ºC)** | 27.7**^c^** ±0.05 | 28.2**^b^**±0.15 | 29.5 **^a^** ±0.18 | 29.3 **^a^** ±0.20 | 27.7**^c^** ±0.08 |
| **TDS (mg L^-1^)** | 215.3**^d^**±1.4 | 213.5**^d^**±4.7 | 1467.5**^c^**±5.6 | 8549.1**^a^**±31.3 | 3778.3**^b^**±24.4 |
| **EC (µS/cm)** | 365.04**^d^**±6.6 | 353.5 **^d^**±4.8 | 2454.4**^c^**±21.3 | 16431.6 **^a^** ± 461.7 | 6306.38 **^a^**±29.1 |
| **Cl^-^ (mg L^-1^)** | 25.51**^d^**±0.1 | 22.6**^d^**±1.3 | 414.4 **^c^**±7.6 | 555.35**^b^**±10.0 | 753.25**^a^**±3.77 |
| **Total Hardness (Ca CO_3_ mg L^-1^)** | 114.7 **^d^**±3.1 | 87.9**^d^**±4.6 | 436.9**^c^**±15.1 | 1194.1**^a^**±22.9 | 673.4**^b^**±15.5 |
| **Ca^++^ (mg L^-1^)** | 26.35**^d^**±0.66 | 16.4**^e^** ±0.53 | 57.5 **^c^**±0.95 | 124.6**^a^**±6.19 | 69.11 **^b^**±2.13 |
| **Mg^++^ (mg L^-1^)** | 11.7**^d^**±0.94 | 11.2**^d^**±1.2 | 70.4**^c^**±3.9 | 216.5 **^a^**±4.2 | 120.2**^b^**±4.07 |
| **Nitrite (NO_2_) mg L^-1^** | 0.11**^c^**±0.007 | 0.20**^c^**±0.01 | 0.84**^b^**±0.19 | 1.49 **^a^**±0.36 | 1.13 **^ab^**±0.18 |
| **Nitrate (NO_3_) mg L^-1^** | 0.08 **^b^** ±0.03 | 0.07 **^b^** ±0.04 | 3.13 **^a^** ±0.56 | 2.95 **^a^** ±0.29 | 0.84 **^b^** ±0.25 |
| **Glyphosate (µg L^-1^)** | 5.431 **^a^** ±0.212  (4.5-6.02) | 0.967**^bc^** ±0.039  (0.8-1.10) | 1.123 **^b^** ±0.021  (1.03-1.19) | 0.813 **^c^** ±0.021  (0.75-0.90) | ND |
| **Malathion (ug L^-1^)** | 4.255 **^a^** ±0.168 (3.80-5.01) | 1.752**^c^**±0.091  (1.40-2.10) | 3.317**^b^**±0.045  (3.15-3.50) | 0.90**^d^**±0.052  (0.70-1.10) | ND |
| **Risk assessment** | | | | | |
| **ECC _glyphosate mg L_^-1^(average=** 0.45) | 0.42 | 0.44 | 0.52 | 0.44 | ND |
| **ECC _malathion mg L_^-1^ (average=** 0.27) | 0.28 | 0.20 | 0.32 | 0.40 | 0.20 |
| **RQ _glyphosate_ (average=** 0.2) | 0.18 | 0.19 | 0.22 | 0.19 | ND |
| **RQ _malathion_ (average=** 0.38) | 0.38 | 0.26 | 0.43 | 0.54 | 0.27 |
| **AEC _glyphosate_** | 0.2331 | | | | |
| **AEC _malathion_** | 0.0738 | | | | |

Means with different superscript letters within the same row are significantly different (P < 0.001); * (P < 0.05), ND= not detected, ECC=expected environmental concentration, RQ= risk quotient, AEC = acute effect concentration

**Table (S3): Formulation and proximate analysis of the experimental diets (g Kg^-1^ total diet):**

| Ingredient | | Chemical composition by proximate analysis (% Dry matter) | |
| --- | --- | --- | --- |
|  |  | Dry matter | 94.22 |
| Fish meal | 300 | Crude protein | 42.01 |
| Soy bean | 350 | Crude lipid | 6.3 |
| Vit&Mineral mix | 3 | Crude fiber | 4.9 |
| Corn starch | 150 | Ash | 7.43 |
| Soybean oil | 25 | Nitrogen free extract | 39.18 |
| Wheat bran | 25 | Gross energy (kcal/kg) | 460 |

**Vitamin and mineral premix** (per kg of mixture) contain the following :15000 IU vitamin A, 1500 IU vitamin D3 , 2.0 mg vitamin E, 2mg vitamin K3, 2.5 vitamin B2, 10 mg vitamin B3, 3 mg vitamin B6, 2 mg vitamin B1, 5 mg vitamin B12, 5.5 mg pantothenic acid, 1 mg niacin, 2 mg folic acid, 100 mg choline, 4 g copper, 300 mg iodine, 30 g iron, 60 g manganese, 50 g zinc, 855.5 g calcium carbonate.

**Table (S4): Probability probit for LC_50_-96 hours for glyphosate and malathion:**

| **Probability** |  | **95% Confidence Limits for Malathion Concentrations** | | | **95% Confidence Limits for Glyphosate Concentrations** | | |
| --- | --- | --- | --- | --- | --- | --- | --- |
|  | LC | Estimate | Lower Bound | Upper Bound | Estimate | Lower Bound | Upper Bound |
| **Probit** | 1 | 0.336 | 0.302 | 0.366 | 1.612 | 1.494 | 1.687 |
|  | 15 | 0.559 | 0.537 | 0.579 | 2.011 | 1.980 | 2.047 |
|  | 30 | 0.647 | 0.628 | 0.666 | 2.169 | 2.121 | 2.243 |
|  | 50 | 0.738 | 0.720 | 0.756 | 2.331 | 2.255 | 2.453 |
|  | 70 | 0.829 | 0.809 | 0.849 | 2.493 | 2.387 | 2.666 |
|  | 90 | 0.960 | 0.936 | 0.986 | 2.727 | 2.576 | 2.974 |
